# The Anatomist’s Ode to His Mistress

**Published:** 1875-04

**Authors:** 


The following poem occasionally is found in print, but usually with some corruptions. It was last published in the *Richmond and Louisville Medical Journal*, but with many errors. It was written by a medical student, now a Professor in New York. As it has never been acknowledged publicly we do not feel at liberty to disclose the writer's name, but this is set up in accordance with a copy in the author's own manuscript.

*THE ANATOMIST'S ODE TO HIS MISTRESS.*

I list as thy heart and ascending aorta
  Their volumes of valvular harmony pour;
And my soul from that muscular music has caught a
  New life 'mid its dry anatomical lore.

O rare is the sound when thy ventricles throb
  In a systolic symphony measured and slow,
While the auricles answer with rythmical sob,
  As they murmur a melody wondrously low!

O thy cornea, love, has the radiant light
  Of the sparkle that laughs in the icicle's sheen;
And thy crystalline lens, like a diamond bright,
  Through the quivering frame of thine iris is seen!

And thy retina, spreading its lustre of pearl,
  Like a far away nebula, distantly gleams
From a vault of black cellular mirrors that hurl
  From their hexagon angles the silvery beams.

Ah! the flash of those orbs is enslaving me still,
  As they roll 'neath the palpebræ, dimly translucent,
Obeying in silence the magical will
  Of the oculo-motor—pathetic—abducent.

O sweet is thy voice, as it sighingly swells
  From thy daintily quivering chordæ vocales,
Or rings in clear tones through the echoing cells
  Of the antrum, the ethmoid, and sinus frontales!

And stately the heave of thy maidenly breast
  As the swell of the billow swift rolling to land;
And as soft the vesicular sigh in thy chest
  As the moan of the ripple that ebbs o'er the sand.

But alas ! 'tis with many forebodings I pen
  Anatomical verses thy beauty to praise,
For I fear me my studies will never again
  Bring the solace they had in my happier days.

Thou hast stolen the charm from my studio dim—
  From dissection I turn with embittering wrath—
Thou has stepped betwixt me and my skeletons grim—
  Ah, lady ! fair lady ! why crossed ye my path !

